# Etiology-Discriminative Multimodal Imaging of Left Ventricular Hypertrophy and Synchrotron-Based Assessment of Microstructural Tissue Remodeling

**DOI:** 10.3389/fcvm.2021.670734

**Published:** 2021-05-25

**Authors:** Filip Loncaric, Patricia Garcia-Canadilla, Ana Garcia-Alvarez, Laura Sanchis, Susana Prat, Adelina Doltra, Eduard Quintana, Daniel Pereda, Hector Dejea, Anne Bonnin, Marta Sitges, Bart Bijnens

**Affiliations:** ^1^Institut d'Investigacions Biomèdiques August Pi i Sunyer, Barcelona, Spain; ^2^Cardiovascular Institute, Hospital Clínic and Universitat de Barcelona, Barcelona, Spain; ^3^Photon Science Department, Paul Scherrer Institut, Villigen, Switzerland; ^4^Eidgenössische Technische Hochschule Zurich, Zurich, Switzerland; ^5^Centro de Investigación en Red de Enfermedades Cardiovasculares (CERCA), Madrid, Spain; ^6^Institució Catalana de Recerca i Estudis Avançats, Barcelona, Spain

**Keywords:** hypertrophic cardiomyopathy, hypertension, myocardial disarray, fibrosis, remodeling, synchrotron, speckle tracking, cardiac magnet resonance

## Abstract

**Background:** Distinguishing the etiology of left ventricular hypertrophy (LVH) is clinically relevant due to patient outcomes and management. Easily obtained, echocardiography-based myocardial deformation patterns may improve standard non-invasive phenotyping, however, the relationship between deformation phenotypes and etiology-related, microstructural cardiac remodeling has not been reported. Synchrotron radiation-based X-ray phase-contrast imaging (X-PCI) can provide high resolution, three-dimensional (3D) information on myocardial microstructure. The aim of this pilot study is to apply a multiscale, multimodality protocol in LVH patients undergoing septal myectomy to visualize *in vivo* and *ex vivo* myocardial tissue and relate non-invasive LVH imaging phenotypes to the underlying synchrotron-assessed microstructure.

**Methods and findings:** Three patients (P1-3) undergoing septal myectomy were comprehensively studied. Medical history was collected, and patients were imaged with echocardiography/cardiac magnetic resonance prior to the procedure. Myocardial tissue samples obtained during the myectomy were imaged with X-PCI generating high spatial resolution images (0.65 μm) to assess myocyte organization, 3D connective tissue distribution and vasculature remodeling. Etiology-centered non-invasive imaging phenotypes, based on findings of hypertrophy and late gadolinium enhancement (LGE) distribution, and enriched by speckle-tracking and tissue Doppler echocardiography deformation patterns, identified a clear phenotype of hypertensive heart disease (HTN) in P1, and hypertrophic cardiomyopathy (HCM) in P2/P3. X-PCI showed extensive interstitial fibrosis with normal 3D myocyte and collagen organization in P1. In comparison, in P2/P3, X-PCI showed 3D myocyte and collagen disarray, as well as arterial wall hypertrophy with increased perivascular collagen, compatible with sarcomere-mutation HCM in both patients. The results of this pilot study suggest the association of non-invasive deformation phenotypes with etiology-related myocyte and connective tissue matrix disorganization. A larger patient cohort could enable statistical analysis of group characteristics and the assessment of deformation pattern reproducibility.

**Conclusion:** High-resolution, 3D X-PCI provides novel ways to visualize myocardial remodeling in LVH, and illustrates the correspondence of macrostructural and functional non-invasive phenotypes with invasive microstructural phenotypes, suggesting the potential clinical utility of non-invasive myocardial deformation patterns in phenotyping LVH in everyday clinical practice.

## Introduction

Distinguishing sarcomere protein gene mutation hypertrophic cardiomyopathy (HCM) from other etiologies of left ventricular hypertrophy (LVH) is clinically relevant, given the association with elevated risk for sudden death, familiar inheritance, and different pharmacological management. The contemporary approach to diagnosing LVH is based on non-invasive multimodality imaging [echocardiography and magnetic resonance (CMR)] – where LV chamber size/shape remodeling, distribution of hypertrophy/fibrosis, and functional alterations form the basis for diagnosis (1, 2). Cardiac mechanics are influenced by the structural and pathophysiological processes underlying the LV remodeling. Therefore, easily obtained, echocardiography-based myocardial deformation patterns, reflecting cardiac mechanics and suggested as highly characteristic for specific etiologies (3), may improve phenotyping when added to the traditional data integration. Nevertheless, the relationship between deformation phenotypes and etiology-related microstructural cardiac remodeling has not been reported. While relatively novel in the field of cardiac imaging, synchrotron radiation-based X-ray phase-contrast imaging (X-PCI) can provide high resolution, three-dimensional (3D) histological information on myocardial microstructure *ex vivo* non-destructively (4–7) – providing a novel approach to visualize complex structural changes related to remodeling in different disease etiologies.

**Figure 1:**
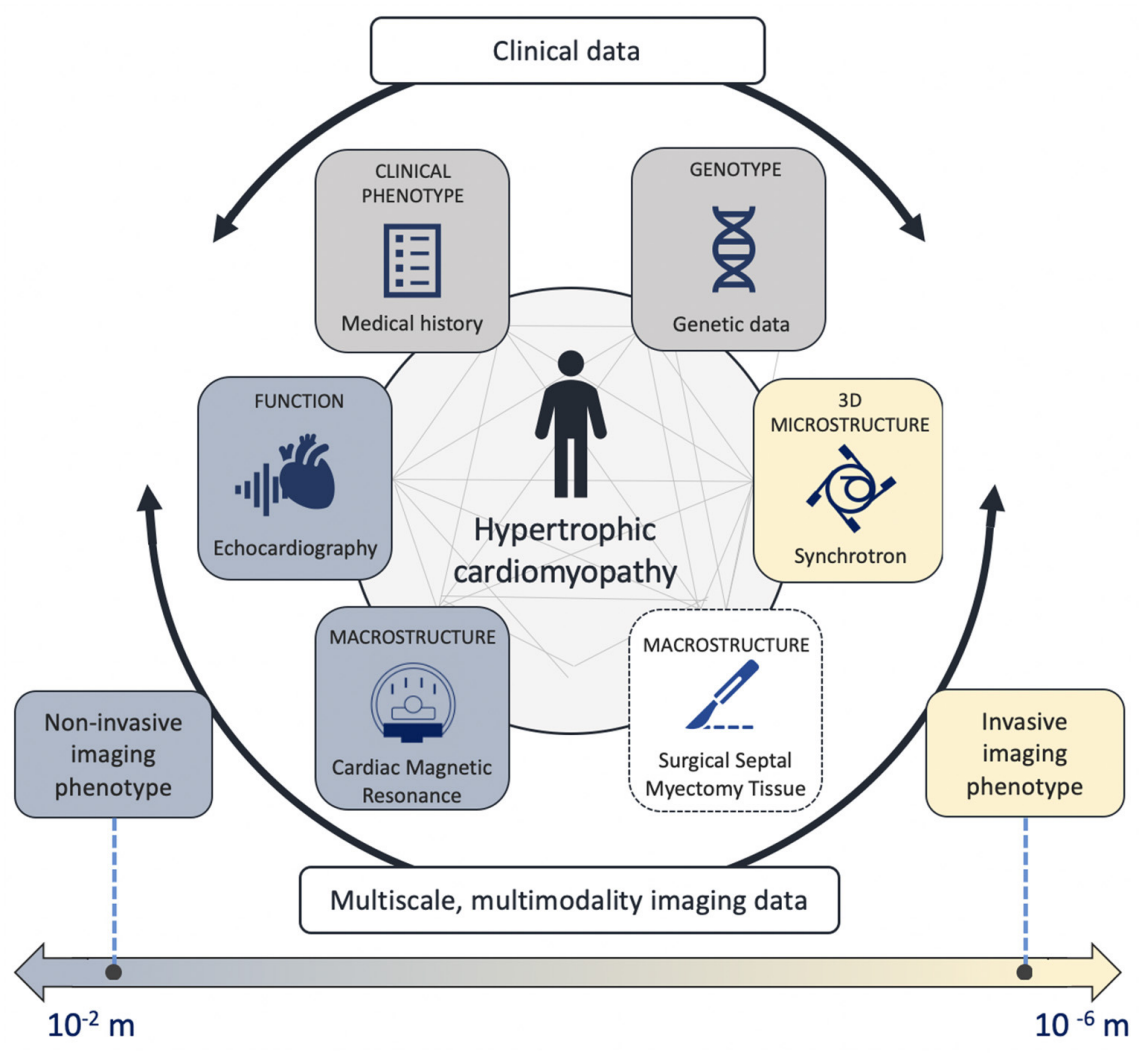
Central figure – The multiscale, multimodality analysis evaluating genetic information, microstructure, macrostructure and cardiac function.

The aim of this pilot study is to apply a multiscale, multimodality protocol in LVH patients undergoing septal myectomy to visualize *in vivo* and *ex vivo* myocardial tissue with non-invasive and X-PCI imaging, respectively, and relate non-invasive LVH imaging phenotypes to the underlying synchrotron-assessed microstructure (Figure 1).

## Methods

### Patients Under Study

The population under study were three patients (P1-3) with obstructive LVH referred for septal myectomy from the familiar cardiomyopathy outpatient clinic. At the moment of inclusion, a clinical interview was performed. Medical history was reviewed and data on demographic characteristics, cardiovascular risk factors, comorbidities, pharmacological treatment was collected. The 5-year HCM risk score was evaluated (8). Upper arm cuff blood pressure measurement in the sitting position was performed directly preceding the echo examination. Genomic DNA was obtained from peripheral blood and analyzed using NGS technology (*MiSeq, Illumina*). The most prevalent gene mutations involved in cardiomyopathies were scanned (i.e., hypertrophic cardiomyopathy (CM), laminopathies, Danon disease, dilatative CM, arrhythmogenic CM, non-compaction CM, Marfan syndrome, long QT syndrome, Holt-Oram, Ehlers-Danlos, and Brrugada syndrome). A generational family pedigree was assessed to explore the genetic origin of disease.

### Echocardiography

All participants underwent a comprehensive transthoracic echocardiographic examination, prior to the surgical myectomy, performed on a commercially available E95 system (*GE, Vingmed Ultrasound, Horten, Norway*) equipped with a 4Vc transthoracic transducer. In addition to full two-dimensional and Doppler echocardiography, additional parasternal short-axis, and 4-chamber, 2-chamber, and 3-chamber apical acquisitions with appropriate frame rates were obtained for speckle-tracking and Tissue Doppler analysis, respectively.

LV and LA volumes were assessed in the apical 4- and 2-chamber views. LV ejection fraction was calculated by using the biplane Simpson method. LA volumes were indexed to the BSA. Cardiac dimensions were measured in appropriate 2D views, as according to current guidelines (9). LV mass was calculated using the linear method and normalized by body surface area, whereas hypertrophy was defined as >88 g/m^2^ in females and >102 g/m^2^ in males (9). Relative wall thickness (RWT) was calculated by dividing the doubled value of the end-diastolic posterior wall thickness with the end-diastolic internal diameter of the LV. The type of LV remodeling was determined based on the RWT and indexed LV mass.

Resting LVOT peak velocity was measured by using continuous-wave Doppler echocardiography, and the LVOT pressure gradient peak estimated by using a simplified Bernoulli equation. The maximal LVOT gradient was defined as the highest recorded gradient, either in rest of during Valsalva maneuver. Pulsed-wave Doppler was performed in the apical 4-chamber view by placing the sample volume at the level of the leaflet tips to obtain mitral inflow velocities. Peak velocity of early (E) and late (A) diastolic filling, E velocity deceleration time and A wave duration were measured, and the E/A ratio calculated. Isovolumic relaxation time (IVRT) was measured as the time difference between aortic valve closure and mitral valve opening as assessed in the five-chamber view using continuous-wave Doppler of the LV outflow tract. Tissue Doppler was used to measure early and late diastolic mitral annular velocity at the septal (e' and a' septal) and lateral (e' and a' lateral) annular sites. Myocardial deformation of the left ventricle (LV) was assessed with speckle tracking echocardiography (STE) on 2D grayscale images obtained from the three apical cardiac views and with tissue Doppler deformation imaging (TDI) in the 4-chamber view – both using GE Echopac software (*GE Medical Systems, version 202.41.0*). The endocardial border was manually marked at end-systole of the LV. A region of interest with six segments was automatically generated. If needed, manual adjustments were performed to achieve optimal tracking. Longitudinal strain curves were generated and end-systolic strain, defined by the aortic valve closure time, measured. The LV global longitudinal strain was calculated by averaging values of the 18 segments. The focus of analysis was the basal and mid septal region, as this is where myocardial tissue is removed during septal myectomy. Regional deformation was therefore explored by placing the region of interest (ROI) in different parts of the interventricular septum. TDI-derived deformation was assessed using color-coded maps of myocardial deformation and by determining ROIs to generate regional TDI-derived longitudinal strain curves of myocardial areas within the adjacent ventricular segment.

### Cardiac Magnetic Resonance Imaging

All cardiac magnetic resonance (CMR) exams were performed in a 3.0 Tesla scanner (Signa Architect, General Electric Healthcare), equipped with a 32-channel chest coil. All images were ECG-triggered and obtained in apnea. Standard short axis SSFP cines were obtained (slice thickness 6–8 mm, 2–4 mm gap) in order to calculate left and right ventricular volumes and function. Additionally, 4-, 3-, and 2-chamber SSFP cines were acquired. A standard phase contrast in-plane flow sequence in a 3-chamber view orientation (or a similar orientation with a good visualization of the LV outflow tract) was acquired in order to assess for the presence of flow obstruction. An additional through-plane phase contrast image was obtained at the point of maximal turbulence; VENC was appropriately adjusted in each case to avoid aliasing. A perfusion sequence in three standard short axis orientations (basal, midventricular and apical) was obtained after administration of a single bolus of gadolinium-based contrast (0.15 mmol/kg). Seven min after contrast administration a standard Look-Locker sequence was acquired and the optimal inversion time selected for each patient. Immediately after, late gadolinium enhancement imaging (inversion recovery gradient echo and PSIR sequences) was acquired using the same imaging planes, slice thickness and spacing as the cine images; inversion time was adjusted during the acquisition, if necessary.

### Surgical Myectomy and Tissue Handling

Septal tissue samples were obtained as a part of the planned surgical procedure performed at the center. Patients underwent myectomy as previously described (10). No additional invasive procedure was performed beyond the ones indicated for the management of the patient's clinical condition. The tissue that was removed by the expert judgement of the cardiac surgeon. The number and size of tissue specimens were dependent on the predisposing patient anatomy. The tissue was initially placed in a vile with a heparin solution, and afterwards fixed and stored in a formalin solution at room temperature in a standard formalin container regularly used in the clinical setting (*DiaPath SafeCapsule 31.7 ml*). Tissue samples were measured and photographed for reference.

### Synchrotron Imaging and Data Analysis

Samples were imported to the TOMCAT beamline at Swiss Light Source (*Villigen, Switzerland*) according to the participating institution's ethical recommendations and the governing international legal requirements. Due to limited available beamtime, only the largest removed pieces of tissue were selected for scanning. The tissue was imaged using X-PCI. A multiscale protocol combining a low-resolution (LR) and a high-resolution (HR) setup (5.8 and 0.65 μm pixel size, respectively) was used0 (7). Briefly, the tissue sample was introduced in a tube with deionized degassed water in order to minimally affect the tissue structural conditions and avoid bubble formation. After positioning the sample on the rotation stage, image acquisition was performed using a 20 keV parallel synchrotron X-ray beam. The sample was first imaged at LR (5,001 projections, exposure time = 30 ms, field of view (FoV) = 11.83 × 3.29 mm^2^, 360° rotation) with a sample-detector distance of 333 cm. X-rays were converted to visible light through a LuAG:Ce 300 μm scintillator and detected by a sCMOS camera (PCO Edge 4.2). LR scans correspond well to traditional histology (11), enabling a non-destructive evaluation of the overall morphology of the septal tissue – allowing visualization of the endocardium, an overview of myocyte organization to locate regional disruptions, and the identification of patches of replacement fibrosis or areas with interstitial fibrosis. Furthermore, LR scans were also used to select several ROIs to be imaged at HR (2,501 projections, exposure time = 220 ms, FoV 1.64 × 1.38 mm^2^, 180° rotation), with a sample-detector distance of 20 cm, a LuAG:Ce 20 μm scintillator and a PCO. Edge 5.5 CMOS detector. Additionally, 50 pre-flat, 50 post-flat and 50 dark images were acquired for flat-field and dark-field corrections of each acquisition. The acquired projections were reconstructed using the Gridrec algorithm. In the case of HR, the single distance phase retrieval method developed by Paganin was applied (12, 13) using specific ring correction (14). Several overlapping scans were acquired to cover the full sample in LR or the full ROI in HR. These scans were later stitched in order to obtain full sample/ROI datasets. HR images enabled assessment of individual myocyte organization, vessels morphology and collagen distribution.

X-PCI datasets were visualized and analyzed with *Fiji* (stitching/cropping) (15) and the open-source software *Ilastik* (collagen segmentation) (16) with the aim of reproducible segmentations of the different microstructural components. Specifically, the pixel classification workflow of *Ilastik* was used for collagen segmentation. Finally, 3D slicer was used to generate the 3D volume renders of segmented collagen (17).

## Results

### Non-Invasive Imaging Phenotypes – Macrostructure and Function

**Figure 2:**
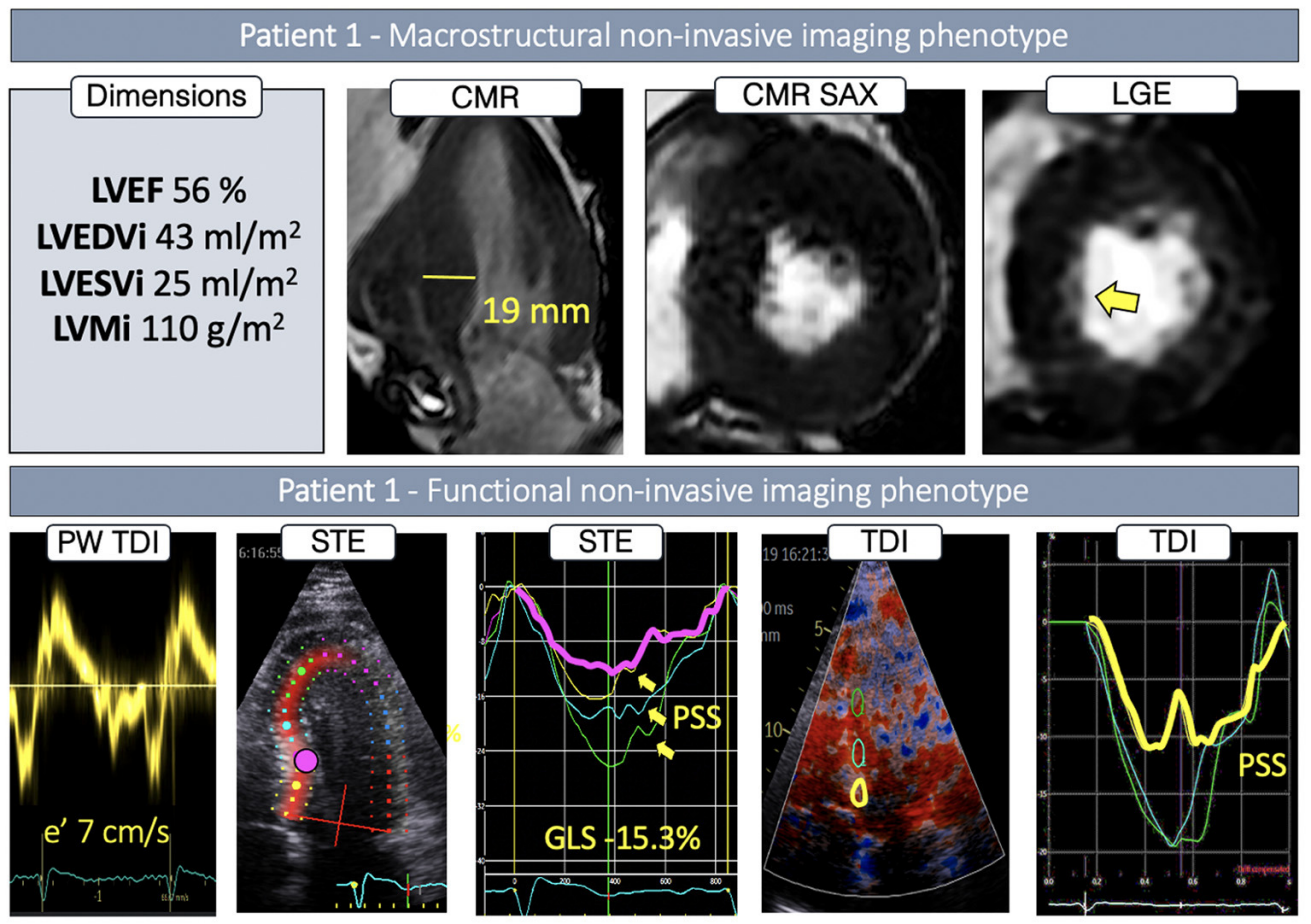
Non-invasive imaging phenotype of P1.

**Figure 3:**
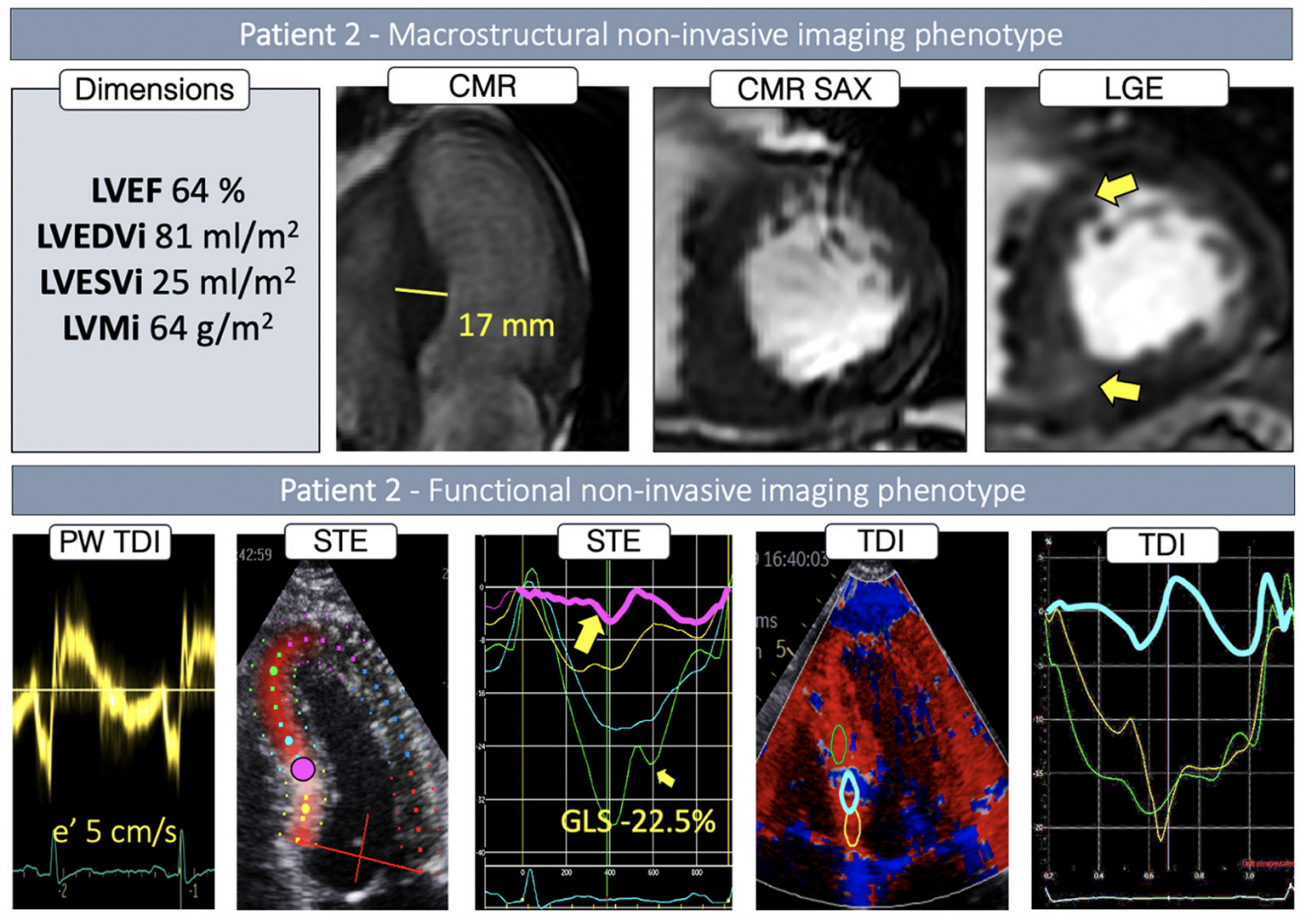
Non-invasive imaging phenotype of P2.

**Figure 4:**
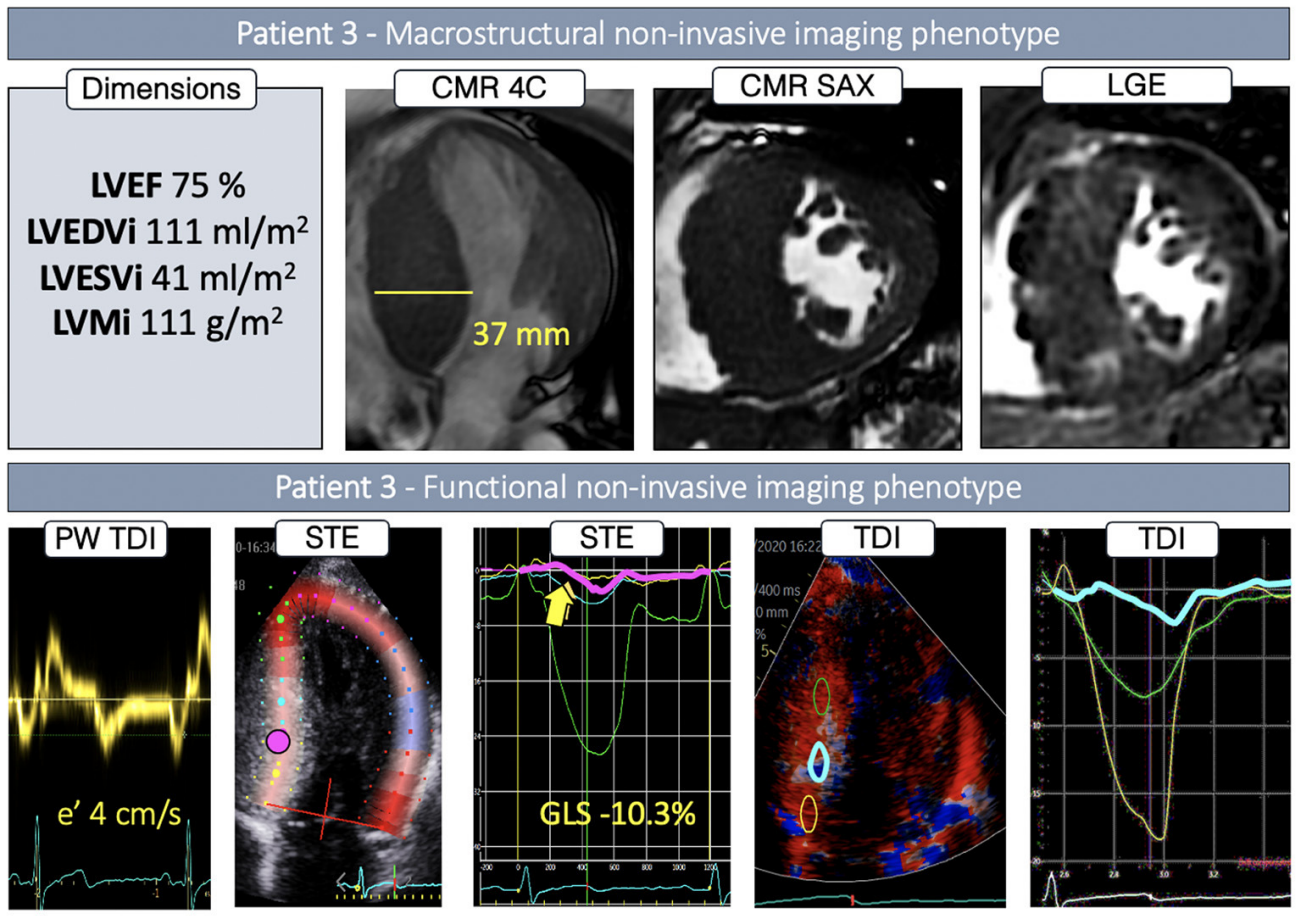
Non-invasive imaging phenotype of P3.

Information on family history of HCM, demographic data and medical history is shown in Table 1, and additional echocardiographic measurements in Supplementary Table 1. All patients had severe symptomatic LV outflow tract obstruction with systolic anterior motion (SAM) of the mitral valve. The non-invasive imaging for P1-3 is illustrated in Figures 2–4.

**Table 1:** Clinical data and medical history.

	**Patient 1**	**Patient 2**	**Patient 3**
Age (years)	64	50	32
Gender	Male	Male	Female
Body mass index (kg/m^2^)	32.0	26.5	22.8
Arterial hypertension	Yes	No	No
Diabetes mellitus	Yes	No	No
Hyperlipidemia	Yes	No	No
Sleep apnea	Yes	No	No
Atrial fibrillation	Paroxysmal	No	No
New York Heart Association Functional Classification	III	III	III
Known family history of HCM	–	Brother had obstructive HCM and underwent septal myectomy	–
Family history of SCD	No	No	No
Cardiac sarcomere protein gene mutations	Negative for tested variants	MYH7	Negative for tested variants
History of unexplained presyncope or syncope	No	No	Yes
History of arrhythmia	No	Non-sustained ventricular tachycardia (2017)	No
5-year HCM risk (%)	2.09	6.25	4.88

P1 had normal LV cavity dimensions, showing concentric LVH combined with pronounced basal anteroseptal hypertrophy (19 mm) (Figure 2), while LGE showed traces of septal intramyocardial and pronounced endocardial fibrosis (Figure 2, first *row, yellow arrow)*. Global longitudinal strain was reduced, whereas regional STE deformation analysis revealed impaired deformation at both the basal, basal/mid and mid septum (Figure 2, *yellow, pink and blue curves*), associated with post-systolic shortening (PSS) (Figure 2, *yellow arrows*). Upon further exploration with TDI, areas with reduced deformation and PSS were identified in the basal septum.

P2 had normal LV dimensions and an asymmetric LVH localized in the inferoseptal region (17 mm) (Figure 3), with focal intramyocardial enhancement in the basal and mid inferior septum and both right ventricular insertion points (Figure 3, first *row, yellow arrows*). While global longitudinal strain was only slightly reduced, septal regional STE deformation analysis showed a heterogeneous deformation pattern: reduced, but normally profiled deformation in the basal segment (Figure 3, *yellow curve*), virtually completely absent deformation on the transition from basal to mid region (Figure 3, *pink curve*), and normalizing deformation toward the apex (Figure 3, *blue and green curves*). Exploration with color-coded TDI and TDI deformation curves confirmed these findings visualizing an isolated area with very abnormal deformation in the transition from basal to mid septum (Figure 3, *blue septal region in the color-coded TDI, blue strain curve*).

P3 had a slightly dilated LV, with an extreme, asymmetric LVH, localized throughout the whole septum (37 mm), and paired with severe enhancement in the septum and inferior interventricular junction (Figure 4). While ejection fraction was supranormal, global longitudinal strain and septal e' velocity was severely reduced. Septal regional STE deformation analysis showed completely abnormal deformation throughout the basal and mid region (Figure 4, *yellow, pink and blue curves*), returning to normal values in the apex (Figure 4, *green curve*). Color-coded TDI and TDI deformation curves, based on smaller, more focused regions of interest, revealed an underlying heterogeneous deformation pattern. Deformation in the basal region was, in fact, normal (Figure 4, *yellow curve*), whereas the transition from basal to mid septum showed completely abnormal deformation (Figure 4, *blue curve*), slowly recovering toward the apical region (Figure 4, *green curve*).

### Invasive Imaging Phenotypes – Microstructure

**Figure 5:**
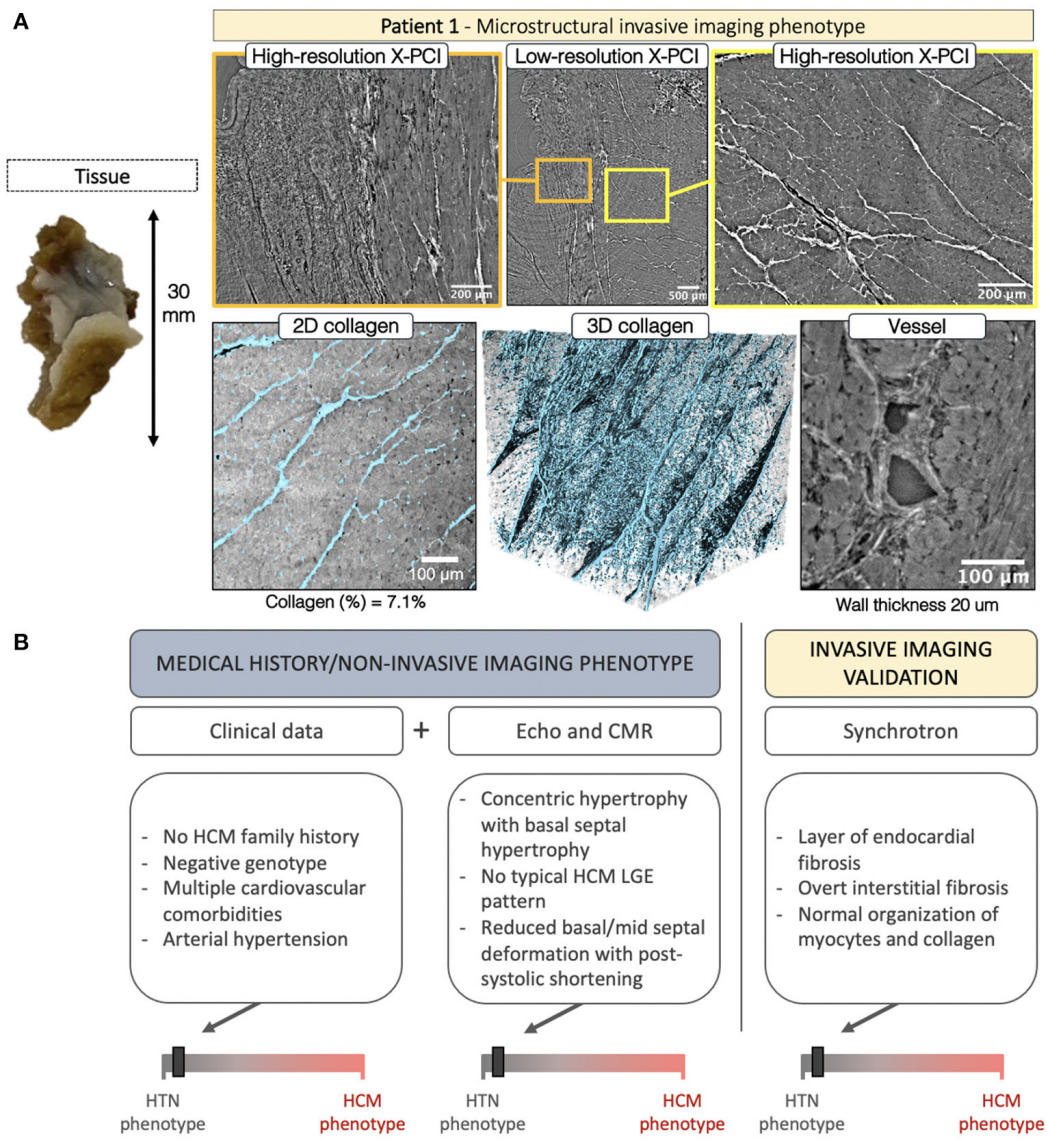
**(A)** Invasive imaging phenotype of P1. **(B)** Clinical data described a patient with no family history of HCM, negative genotype, and a burden of cardiovascular comorbidities, including arterial hypertension. The non-invasive imaging concurred with the HTN clinical phenotype showing basal septal hypertrophy with no typical HCM LGE pattern and a characteristic hypertensive spatiotemporal deformation pattern in the basal and mid septum coupled with post-systolic deformation. The invasive imaging confirmed the non-invasive-imaging-based HTN phenotype with findings of normal myocyte, collagen and blood vessel organization.

After surgical removal in P1, the septal tissue showed a smooth endocardial fibrotic layer which could be visualized with LR, and the underlying collagen organization with HR X-PCI (Figure 5A, *orange frame*, Supplementary Video 1, 2). HR revealed extensive interstitial fibrosis surrounding normally arranged myocytes (Figure 5A, *yellow frame*). The segmented 3D collagen distribution was visualized (Figure 5A, second *row, collagen shown in blue*, Supplementary Video 3), demonstrating extensive (quantified at 7.1% in the selected volume), but spatially normally organized, fibrosis. A blood vessel is shown with normal dimensions, normal perivascular collagen, and mild wall hypertrophy (20 μm). The integration of clinical data for P1 is shown in Figure 5B.

**Figure 6:**
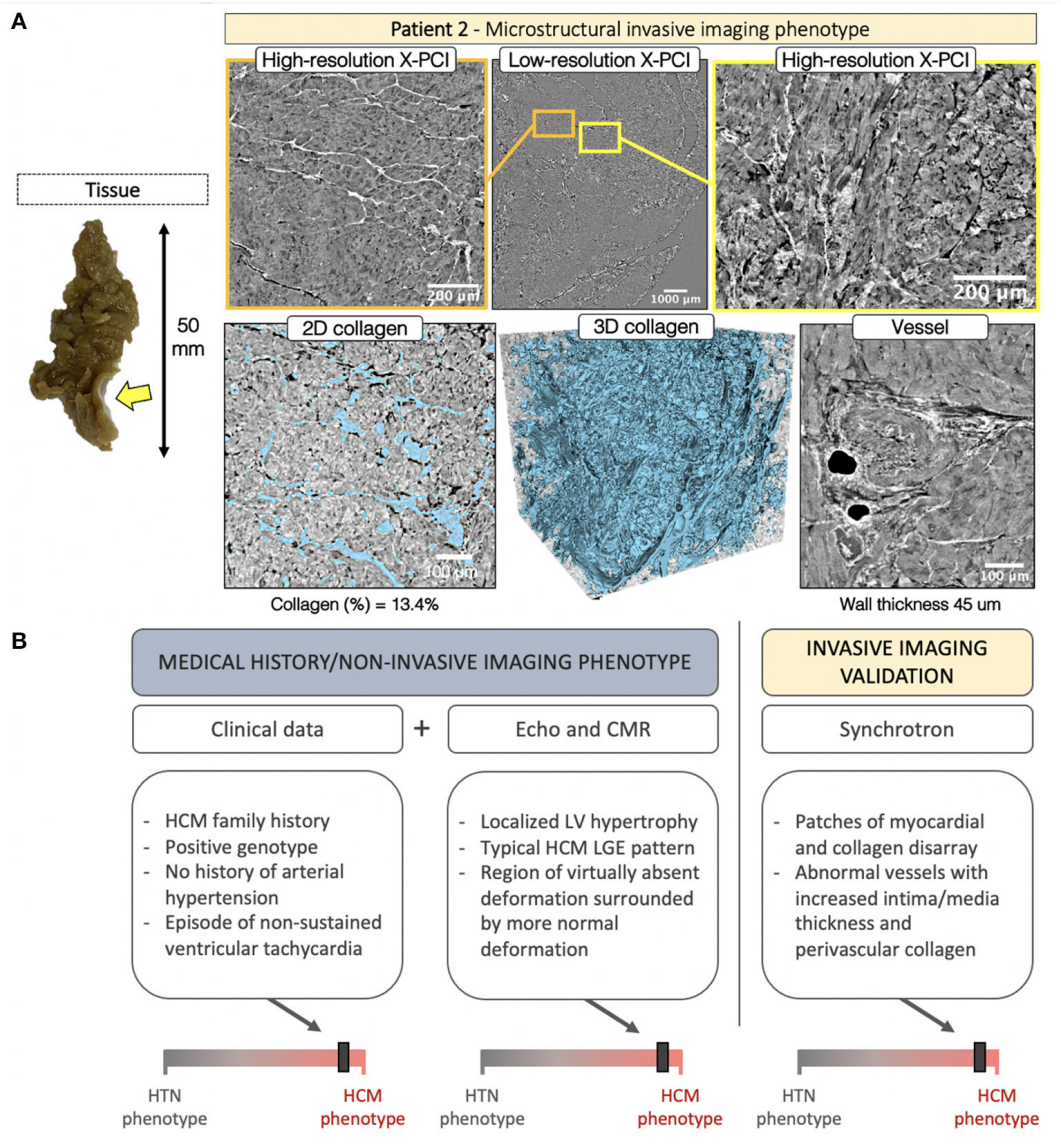
**(A)** Invasive imaging phenotype of P2. **(B)** Clinical data described a patient with family history of obstructive HCM, history of arrhythmic episodes, and a positive genotype. Here, non-invasive imaging confirmed the clinical HCM phenotype. Although localized basal septal hypertrophy can also be seen in hypertension, the deformation pattern was characteristic of HCM, and the LGE findings concurred. Invasive imaging validated the non-invasive-imaging-based HCM remodeling phenotype with findings of myocardial disarray, abnormal collagen organization and hypertrophied blood vessels.

In P2 the septal tissue revealed a localized (likely related to the SAM impact) fibrotic patch on the endocardial side (Figure 6A, *yellow arrow*). LR revealed regions of normal myocardial organization (Figure 6A, *orange frame*) alternated with patches of myocyte disarray (Figure 6A, *yellow frame*, Supplementary Video 4). Disarray was further explored with HR. Visualization of the 3D collagen distribution (Figure 6A, second *row, collagen shown in blue*) showed increased fibrosis (13.4%), highly disorganized, except in the smaller regions of normal organization (Figure 6A, *yellow arrow*, Supplementary Video 5). The vasculature revealed abnormal vessels with intima/media hypertrophy (45 μm) and increased perivascular collagen. The integration of data for P2 is shown in Figure 6B.

**Figure 7:**
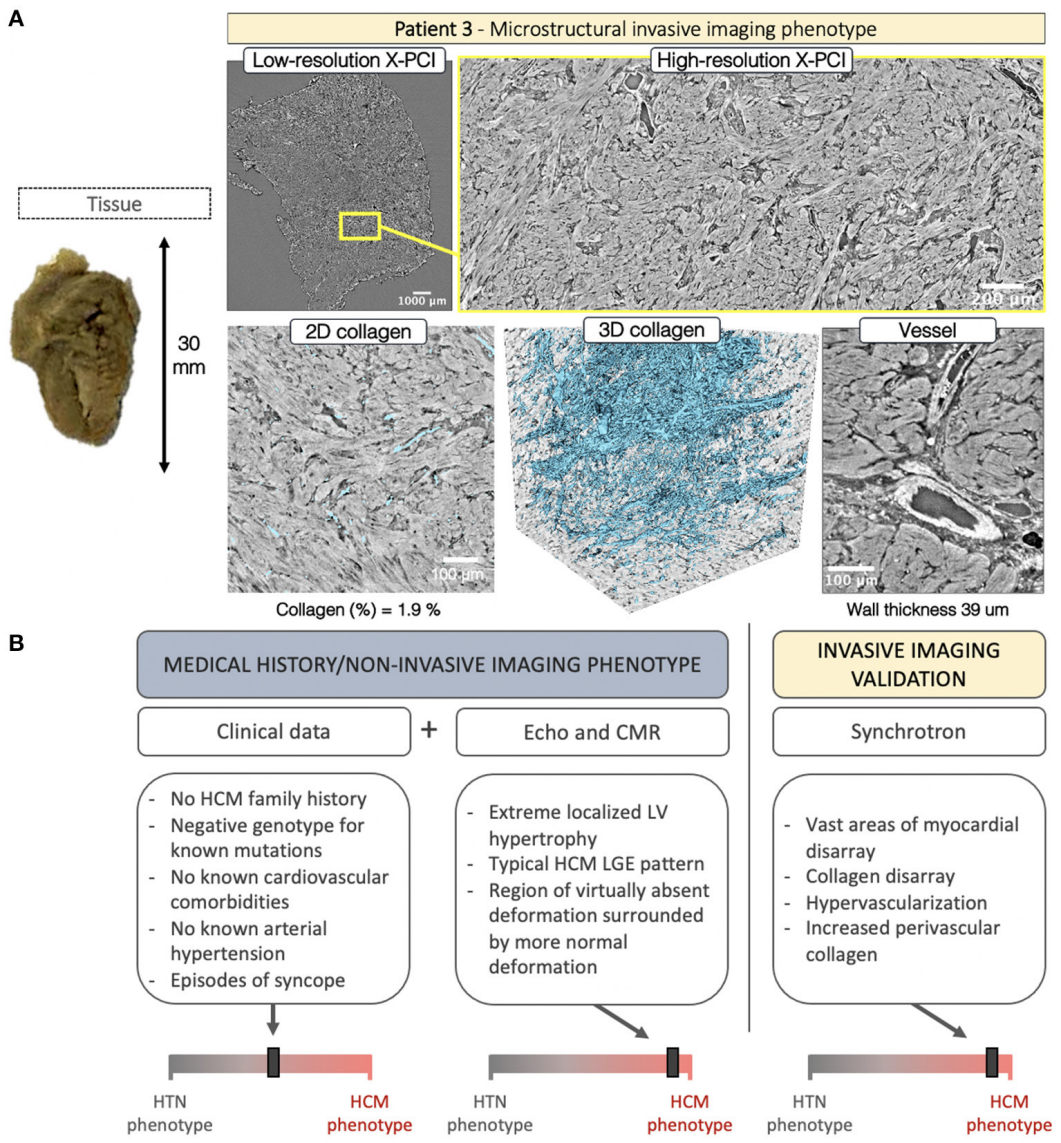
**(A)** Invasive imaging phenotype of P3. **(B)** Clinical data was inconclusive, revealing negative family history of HCM, lack of cardiovascular comorbidities, a history presyncope, and a genotype negative for the most common mutations associated with HCM. Here, non-invasive imaging played a crucial role in revealing the etiology – showing extreme wall thickening in the septal region, with a characteristic HCM deformation pattern, and severe LGE in the septum. Invasive imaging confirmed the non-invasive-imaging HCM remodeling phenotype with findings of vast myocardial disarray, collagen disarray, hypervascular tissue and hypertrophied blood vessels.

Septal myocardial samples from P3 showed no pronounced fibrotic patch visible on the endocardial side. LR, further enhanced with HR, revealed hypertrophied myocytes with overt myocyte disarray, interlaced with small patches of normal organization (Figure 7A, *yellow frame*, Supplementary Video 6). In the acquired tissue sample, collagen was not increased (1.9%), but showed spatial disarray (Supplementary Video 7). Pronounced hypervascularization was noted throughout the sample with an increased number of blood vessels, combined with wall hypertrophy (39 μm) and increased perivascular collagen.

## Discussion

In this pilot study we demonstrate a unique multiscale, multimodality protocol to visualize *in vivo* and *ex vivo* myocardial tissue with non-invasive and X-PCI imaging. We report a novel approach to visualize 3D cardiac microstructure in LVH and relate the findings to the etiology-discriminative interpretation of non-invasive imaging data. The findings are hypothesis-generating, providing insights about the relationship of myocyte and connective tissue matrix spatial disorganization and myocardial deformation – thus suggesting the potential clinical utility of deformation patterns in phenotyping left ventricular hypertrophy.

### The Functional Consequences of Myoarchitectural Abnormalities

X-PCI has proven as a powerful tool for visualizing cardiac microstructure in cardiac biopsies of rat models, human fetal hearts, as well as in endomyocardial biopsies of heart transplantation patients. The modality enables imaging up to the scale of an individual myocyte, demonstrating feasibility of quantification of fiber orientation, vessels and collagen from multiscale 3D datasets; enabling multiresolution, 3D, quantitative *ex vivo* analysis of cardiac microstructure, without the need for artifact prone slice-processing that strains histological/microscopic reconstruction (4, 5, 7, 18). In our study, X-PCI and the subsequent analysis - applying machine-learning solutions to provide automated, 3D segmentations of myocardial structure - enabled a novel way to visualize and quantify the complex microstructural abnormalities that inherently influence cardiac mechanics in LVH.

The histological findings of myocyte (19, 20) and connective tissue matrix (21, 22) disorganization in HCM has evoked research relating abnormal myocardial architecture to LV function. A transgenic HCM mouse model demonstrated decreased sarcomere length and impaired systolic shortening in regions of myocardial hypertrophy (23). In human hearts non-invasive imaging, histology, and *in vitro* experiments suggested an association between disarray, fibrosis, active contraction *in vitro*, and STE-derived deformation *in vivo* (24, 25). Similarly, diffusion tensor CMR and CMR-derived strain rate imaging inferred intramural disarray correlated with both active and passive myocardial function (26). The intrinsic contractile dysfunction can be recognized with deformation imaging even at the early disease stages, before onset of LVH (27). This described relationship between abnormal myoarchitecture and cardiac mechanics in HCM is captured by characteristic deformation patterns, easily obtained in everyday workflow, and potentially clinically useful in the process of distinguishing LVH etiologies, especially when integrated with remaining clinical and imaging findings.

### The Challenge of Distinguishing Disease Etiologies

Clinical practice relies on interpretation of available clinical information for diagnosis in LVH – medical history and physical examination, genetic analysis for the most frequent mutations, and insights gained from non-invasive cardiac imaging (28). The challenge for a genetic diagnosis is the considerable genetic heterogeneity of HCM, where the underlying genetic cause is only found in a percentage of patients fitting the phenotype (29). In our analysis, P3 had a “textbook” microstructural, macrostructural, and functional HCM phenotype, however, the genotyping approach was unsuccessful in identifying the causative genetic mutation strongly suggested to be present. Differentiating HCM from hypertensive heart disease is a process based on integrating findings from clinical history and multimodal non-invasive imaging - the distribution of LVH and LGE, frequency of LV outflow tract obstruction, severity of longitudinal dysfunction, functional asynchrony and deformation heterogeneity (2). Such integration is shown in Figures 5B, 6B, 7B. In this analysis, echo-based regional deformation patterns, reflective of the influence of structural and pathophysiological processes in LV remodeling on cardiac mechanics, are easily accessible and of potential clinical use (3).

The echocardiographic finding of basal septal hypertrophy has been shown to be a morphological marker of increased afterload in arterial hypertension (30, 31) Here intra-ventricular heterogeneity is the consequence of heterogeneous wall stress distribution in response to elevated blood pressure. In an average heart, the septum has a greater radius of curvature compared to the free wall (32, 33), leading to a disproportionately higher wall stress in the basal parts in the setting of high systemic pressure (33). This results with an imbalance between locally developed force and wall stress, and, consequently, decreased local deformation and PSS. Thus, in HTN, prolonged exposure to increased afterload can result in compensatory localized basal septal hypertrophy in an attempt to normalize wall stress and maintain deformation (3), and ultimately lead to LV outflow obstruction (34). Localized hypertrophy in HTN, as seen in P1 and opposed to P2, was associated with microstructurally described overt interstitial fibrosis (not clearly inferred by LGE), showing organized 3D structure of the collagen surrounding normally arranged myocytes. These microstructural findings support the hypertensive etiology of the hypertrophy, especially when combined with the HTN-related deformation pattern - reduction in peak systolic strain (30, 35) and PSS occurring in the basal and mid-septum (36).

Another type of intra-ventricular heterogeneity can be seen in HCM, where, as compared to the heterogeneity of loading in HTN, the heterogeneity is in the tissue structure itself. In our study, X-PCI of HCM myocardial tissue revealed both myocyte and connective tissue matrix 3D disorganization or disarray. The heterogeneous tissue and compensatory hypertrophy leads to high variability in regional myocardial wall thickness and characteristic regional contractile heterogeneity (37–40). The structural finding of basal septal hypertrophy in HCM commonly overlaps with that seen in HTN (e.g., P1 vs. P2), however, with clearly different patterns of deformation. In P2 and P3, we noted a heterogeneous septal deformation pattern, with localized parts of the septum showing virtually absent deformation, unlike that seen in HTN, while surrounding regions show normal deformation pattern (with/without reduced amplitude).

Regional longitudinal strain is still burdened by reproducibility and inter-vendor variability (41–43). Nevertheless, regional spatiotemporal strain patterns contain important diagnostic information (3, 41), and remain consistent despite underlying variability in inter-observer segmentations and regional peak strain values (44). The results of this pilot study are hypothesis-generating, suggesting non-invasive deformation phenotypes are associated with etiology-related myocyte and connective tissue matrix 3D disorganization, thus inferring the potential clinical value of deformation patterns in everyday clinical analysis and phenotyping LV hypertrophy.

### Scientific and Clinical Implications

The ability to explore microstructural organization is essential for understanding myocardial mechanics and resolving the etiology of non-invasive imaging phenotypes in LV hypertrophy. In contemporary translational research, electro-mechanical computational models of the heart integrate multiscale and multimodal imaging, and apply novel methods of data extraction from large datasets and across different resolutions, with the aim toward deciphering mechanistic descriptors of personalized structure and function (45). Detailed information on fiber orientation and fibrosis organization can be integrated to these models (46), a concept particularly relevant in HCM (47). Previously, fiber structure information has been extracted from diffusion tensor imaging (at low resolution) or localized microscopy (48), but this may be advanced with high resolution, 3D X-PCI data. Derived information about disease-specific patterns could help relate structural and dynamic features measured *in vivo* with high-resolution characterization of microstructure *ex vivo*, enabling personalized modeling of cardiac biomechanics, potentially bringing incremental insights to disease pathophysiology and tailoring risk assessment (49).

In a more clinical perspective, continuing evidence point out the inconsistencies of the single sarcomere gene hypothesis in HCM, suggesting the need to incorporate the influences of numerous disease modifiers, each exerting a small effect on phenotype expression (50–52). Here, the opportunity to explore the structure-function relationship, through the insights of combined *in vivo* and *ex vivo* imaging is highly relevant. Applying this methodology enables quantification of myocardial structure abnormalities potentially associated to sarcomere protein gene mutations and clinical risk, even in patients with no signs of macrostructural remodeling when assessed with traditional non-invasive imaging. In comparison to X-PCI, histology is a destructive imaging method, relying on tissue preparation – slicing and staining, and of limited (2D) analysis. Multiscale analysis with CMR may provide better soft tissue contrast, however, at much lower resolutions and/or prolonged scan time. Furthermore, CMR-based techniques rely on indirect measures of myocardial structure, through the use of contrast agents or diffusion tensor imaging, whereas with X-PCI we can directly measure myocardial structure based on changes in X-ray intensity and phase. On an important note, X-PCI is currently a research methodology, and as such it is linked to synchrotron facilities with limited accessibility. Integration of X-PCI technology in traditional hospital CT machines is for now still not feasible (53), but the developments in compact synchrotrons (54) and grating based X-PCI technology (55) may lead to a translation of this methodology towardz clinical, *ex vivo* use – particularly for imaging tissue biopsies. Application of these tools has already been suggested – for clinically relevant topics such as the assessment of *ex vivo* endomyocardial tissue in heart transplantation patients to assess graft rejection with more reproducibility (56), or assessment *in vivo* to clarify ambiguous findings in traditional mammography (57).

### Limitations

Our pilot study consisted of a detailed, multi-modality *in vivo* and *ex vivo* analysis of a small sample, with the goal of applying existing X-PCI technology to the field of cardiac imaging to provide a novel visualization of microstructural organization in LVH, and to generate a hypothesis of structure/function relations by linking these invasive findings to non-invasive imaging phenotypes. Nonetheless, no causation can be claimed based on these initial results. The results motivate larger patient cohorts to enable statistical analysis/group comparison, as well as the assessment of reproducibility of deformation patterns (44).

All imaged tissue samples were derived from surgical myectomy; therefore, the sample size was limited and potentially not representative of the heart as a whole. However, septal tissue has consistently demonstrated structural abnormalities in prior studies (22, 24, 25, 52).

## Conclusions

High-resolution, 3D X-PCI provides novel ways to visualize myocardial remodeling in excised myectomy tissue, and illustrates the correspondence of macrostructural and functional non-invasive phenotypes with invasive microstructural phenotypes, suggesting the potential clinical utility of non-invasive myocardial deformation patterns in phenotyping left ventricular hypertrophy. A larger patient cohort could enable statistical analysis of established differences and the assessment of the reproducibility of deformation patterns.

## Data Availability Statement

The data presented in the study are deposited in the PSI Public Data Repository, accession number http://doi.psi.ch/detail/10.16907%2Fb97cdb87-83be-4176-87f4-2e89679ff333.

## Ethics Statement

The studies involving human participants were reviewed and approved by Ethics Committee of the Hospital Clinic of Barcelona. The patients/participants provided their written informed consent to participate in this study.

## Author Contributions

FL: conception, design, analysis and interpretation of data, and drafting of the manuscript. PG-C: analysis and interpretation of data, drafting of the manuscript, revising for important intellectual content, and final approval of the manuscript submitted. AG-A, HD, and AB: conception and design, revising for important intellectual content, and final approval of the manuscript submitted. LS, SP, AD, EQ, and DP: revising for important intellectual content and final approval of the manuscript submitted. MS and BB: conception and design, interpretation of data, revising for important intellectual content, and final approval of the manuscript submitted. All authors contributed to the article and approved the submitted version.

## Conflict of Interest

The authors declare that the research was conducted in the absence of any commercial or financial relationships that could be construed as a potential conflict of interest.

## Supplementary Material

Supplementary Video 1 Patient 1 - Top to bottom scan of a subvolume of the HR X-PCI Paganin reconstructed dataset.

Supplementary Video 2 Patient 1 - Top to bottom scan of a subvolume of the HR X-PCI Paganin reconstructed dataset.

Supplementary Video 3 Patient 1 – A 3D visualization of the collagen organization.

Supplementary Video 4 Patient 2 - Top to bottom scan of a subvolume of the HR X-PCI Paganin reconstructed dataset.

Supplementary Video 5 Patient 2 – A 3D visualization of the collagen organization.

Supplementary Video 6 Patient 3 - Top to bottom scan of a subvolume of the HR X-PCI Paganin reconstructed dataset.

Supplementary Video 7 Patient 3 – A 3D visualization of the collagen organization.

Supplementary Figure 1 Photographs of tissue collected after surgical myectomy.

Supplementary Table 1 Echocardiographic measurements.

Supplementary Table 2 Measurements of the septal myectomy tissue samples.
