# Supplementary material for: Etiology-Discriminative Multimodal Imaging of Left Ventricular Hypertrophy and Synchrotron-Based Assessment of Microstructural Tissue Remodeling
Source: Front Cardiovasc Med. 2021 May 25;8:670734. doi: 10.3389/fcvm.2021.670734 (PMC8185228; doi:10.3389/fcvm.2021.670734)

**Table S1** Echocardiographic measurements

|  |  | Patient 1 | Patient 2 | Patient 3 |
| --- | --- | --- | --- | --- |
| Echo | LV ejection fraction (%) | 56 | 64 | 75 |
|  | Longitudinal strain - 4-chamber view (%) | 18.1 | 22.2 | 12.7 |
|  | Longitudinal strain - 2-chamber view (%) | 17.7 | 20.6 | 11.2 |
|  | Longitudinal strain - 3-chamber view (%) | 17.5 | 19.3 | 12.4 |
|  | LV global longitudinal strain (%) | 17.8 | 20.7 | 12.1 |
|  | LV basal septal longitudinal strain (%) | 15.98 | 12.63 | 1.22 |
|  | LVOT gradient (mmHg) | 136 | 50 | 49 |
|  | Systolic anterior motion of the mitral valve | + | + | + |
|  | EA ratio | 0.64 | 0.57 | 1.71 |
|  | E deceleration time (ms) | 263 | 369 | 225 |
|  | Fused EA | Yes | Yes | No |
|  | A duration (ms) | 164 | 167 | 131 |
|  | IVRT (ms) | 97 | 119 | 105 |
|  | Septal mitral annulus e’ (cm/s) | 7 | 5 | 4 |
|  | Lateal mitral annulus e’ (cm/s) | 9 | 7 | 7 |
|  | SAPSE | 15 | 14 | 10 |
|  | MAPSE | 16 | 20 | 12 |
|  | TAPSE | 28 | 30 | 28 |

**Table S2** Measurements of the septal myectomy tissue samples

|  | P1 | P2 | P3 |
| --- | --- | --- | --- |
| N | Dimensions (mm) | Dimensions (mm) | Dimensions (mm) |
| 1 | 30x20x6* | 50x20x10* | 30x25x10* |
| 2 | 20x10x4 | - | 30x17x7 |
| 3 | 20x10x10 | - | 30x12x5 |
| 4 | 20x15x3 | - | 20x12x6 |
| 5 | 10x10x3 | - | 20x12x5 |
| 6 | 15x10x4 | - | 20x10x6 |
| 7 | 15x10x4 | - | 20x3x3 |
| 8 | 8x5x5 | - | 10x9x5 |
| 9 | 7x5x3 | - | 13x5x3 |
| 10 | 7x5x3 | - | 14x4x4 |
| 11 | 8x4x3 | - | 9x5x3 |
| 12 | 8x4x4 | - | 12x6x4 |
| 13 | 7x4x3 | - | 7x5x3 |
| 14 | 17x1x1 | - | 6x4x3 |
| 15 | - | - | 7x4x2 |
| 16 | - | - | 7x5x3 |
| 17 | - | - | 6x4x3 |
| 18 | - | - | 5x4x2 |
| 19 | - | - | 9x3x2 |
| * pieces of tissue selected for X-PCI scanning and histological analysis | | | |

**Figure S1** Photographs of tissue collected after surgical myectomy


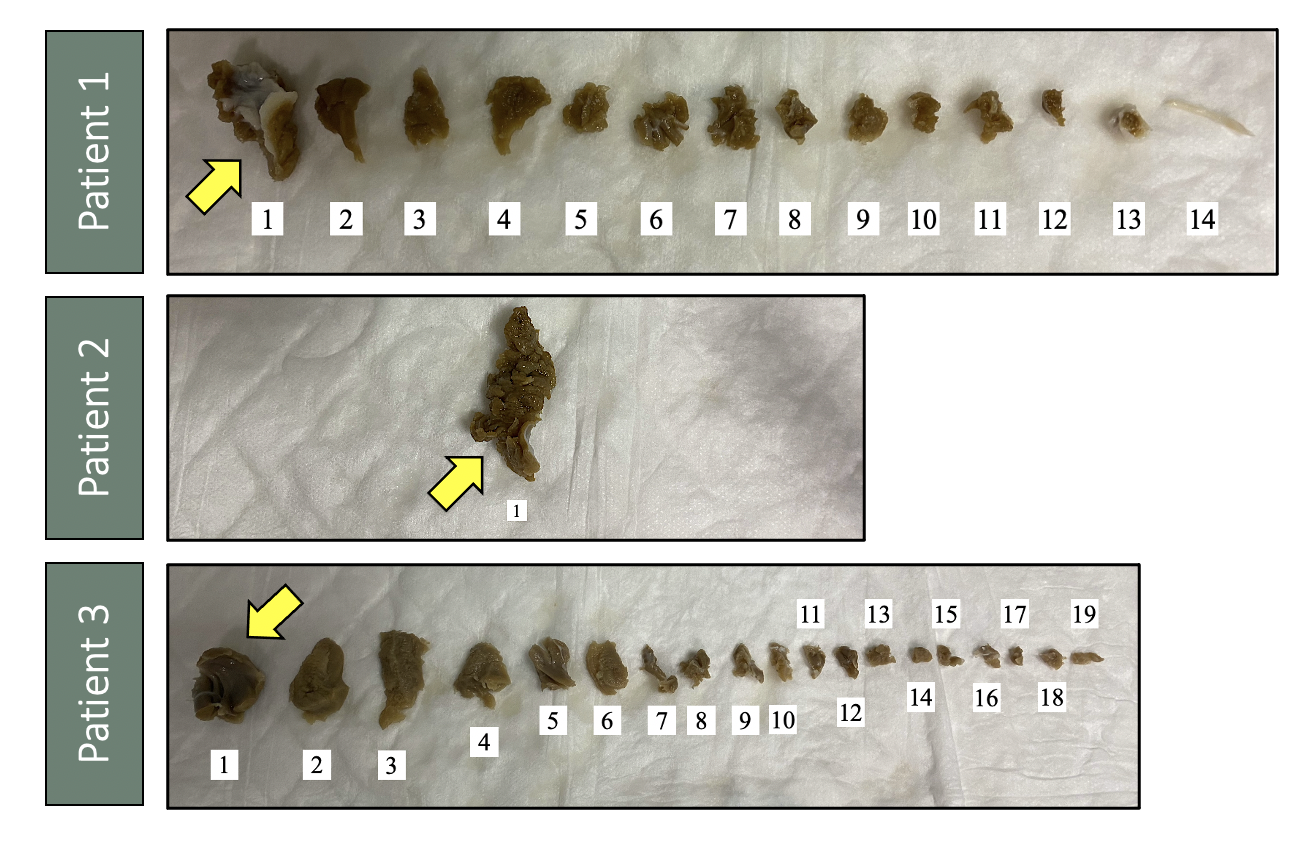

Supplement: Supplementary Figure 1 — Photographs of tissue collected after surgical myectomy. [file Data_Sheet_1.docx]
